# Selenium Nanoparticles in Cancer Therapy: Unveiling Cytotoxic Mechanisms and Therapeutic Potential

**DOI:** 10.1002/cnr2.70210

**Published:** 2025-06-02

**Authors:** Sumaira Anjum, Mariam Hashim, Maham Imran, Sundus Babur, Sanniah Adnan, Christophe Hano, Wisam Nabeel Ibrahim

**Affiliations:** ^1^ Department of Biotechnology Kinnaird College for Women Lahore Pakistan; ^2^ Laboratoire de Biologie Des Ligneux et Des Grandes Cultures INRAE USC1328, Eure & Loir Campus, University of Orleans Chartres France; ^3^ Department of Biomedical Science College of Health Sciences, QU Health, Qatar University Doha Qatar

**Keywords:** cancer, in vitro, oxidative stress, ROS, selenium nanoparticles, selenoproteins

## Abstract

**Background:**

Cancer represents a complex group of diseases characterized by abnormal cell proliferation, invasion, and metastasis. These features pose significant challenges to conventional therapeutic approaches, necessitating the development of more targeted and effective treatment strategies.

**Objective:**

This review aims to explore the potential of selenium nanoparticles (SeNPs) as a novel therapeutic tool in cancer treatment, emphasizing their cytotoxic mechanisms and advantages over conventional therapies and other nanoparticles.

**Methods:**

The review synthesizes findings from recent studies investigating the therapeutic properties of SeNPs in cancer models. Emphasis is placed on their ability to selectively target malignant cells, modulate redox status, and influence tumor‐associated cellular processes such as autophagy and microRNA regulation.

**Results:**

SeNPs demonstrate intrinsic antioxidant properties that counteract oxidative stress commonly observed in cancer cells. They modulate critical cellular pathways and exhibit selective toxicity, damaging cancer cells while sparing healthy tissues. Additionally, their biocompatibility and capacity to deliver therapeutic agents contribute to improved safety and efficacy compared to other nanoparticle platforms.

**Conclusion:**

Selenium nanoparticles hold significant promise as a next‐generation cancer treatment modality. Their dual function—serving as both therapeutic agents and drug delivery vehicles—positions them as a powerful tool in precision oncology. By minimizing off‐target effects and enhancing targeted drug delivery, SeNPs have the potential to advance the landscape of cancer theragnostics.

## 1 Introduction

Over the past three decades, the evolution of nanotechnology has not only catalyzed but also fundamentally transformed the landscape of pharmaceutical research and drug development. This transformative journey has led to the exploration of novel pathways in disease pathophysiology and opened unprecedented vistas in therapeutic interventions [1, 2, 3]. At the heart of this revolution lies nanotechnology, a field centered on manipulating and utilizing materials at the nanoscale, where dimensions are smaller than 100 nm. Within this domain, a rich tapestry of nanomaterials has emerged, each offering unique properties and functionalities that hold immense promise in biomedical applications. Polymers, dendrimers, liposomes, and an array of metal nanoparticles—spanning the likes of silver, gold, cerium, copper, europium, iron, selenium, titanium, yttrium, and beyond—have emerged as versatile platforms for the design and delivery of therapeutics [4, 5, 6, 7]. Nanoparticles (NPs) can be readily recognized by their small size, vast expanse of surface, charge distribution on the surface, surface chemistry, soluble content, and multipurpose use. NPs have successfully delivered medicinal chemicals, demonstrating their effectiveness as carriers of medicines. Nanomedicine is the application of nanotechnology‐based techniques and approaches in clinical practice and medical research to treat, diagnose, and regulate biological structures [7]. NPs increase the permeability of hydrophilic compounds, peptides, protein vaccinations, siRNA, miRNA, DNA, and other biological treatments, as well as the therapeutic efficacy of ionized pharmaceuticals. The drug delivery system is significantly more adaptable and can deliver only to the target spot when the surface of the nanoparticles has been modified with targeting ligands [8, 9]. Among the pantheon of metal nanoparticles, selenium nanoparticles (SeNPs) stand out as a subject of intense investigation and burgeoning interest.

Selenium, an essential trace element intricately woven into the fabric of human biology, orchestrates a symphony of physiological processes, ranging from metabolic homeostasis and thyroid function to DNA synthesis and immune defense [10]. Its utility extends beyond the confines of biology, finding applications in diverse industrial and commercial sectors owing to its unique chemical and physical properties, including a low melting point and high photoconductivity [11]. Selenium, a component of selenium‐containing proteins and seleno‐compounds in human tissues, is necessary for metabolism, thyroid hormone production, DNA synthesis, reproduction, and defense against oxidative stress and pathogens. It finds use in a variety of commercial and industrial applications. Its low melting point and high photoconductivity make it a suitable catalyst for organic hydration and oxidation reactions [12].

Yet, selenium's remarkable antioxidant prowess, mediated through enzymes such as glutathione peroxidase and seleno‐catalysts, underscores its significance in health and disease [13, 14, 15, 16, 17]. In the realm of oncology, selenium has emerged as a potent ally, demonstrating the ability to selectively induce apoptosis in cancer cells while sparing their healthy counterparts [18, 19, 20, 21]. As a result, the concentration, redox state, and kind of Se molecule exploited have a major impact on pharmacological action and cytotoxicity. Supra‐nutritional selenium (Se) levels have been shown to reduce the incidence of various malignancies, including lung, prostate, and colorectal cancer [21, 22, 23, 24]. It inhibits the G2/M proliferation cycle and induces premature cell death in cancer cells via the mitochondrial route [9, 25]. This dichotomy of selenium, acting as both a guardian against oxidative stress at physiological levels and a harbinger of cytotoxicity at supra‐nutritional doses, underscores the delicate balance required for its therapeutic utility [26]. A deficiency in selenium within the diet can precipitate systemic failures, particularly affecting critical organs such as the liver and the hemolytic systems. Insufficient selenium levels are implicated in a spectrum of disorders, notably including Kashin–Beck disease, neurological impairments, and chronic degenerative conditions [27, 28, 29].

Selenium (Se) is a unique trace element known for its incorporation into selenoproteins, enabling it to exert diverse therapeutic effects. Certain selenoproteins, crucial for enzymatic functions, necessitate selenocysteine in their active sites [30, 31, 32]. Numerous studies indicate that selenium (Se) exhibits inadvertent antioxidant activity by stimulating and replenishing vitamin C and Q10 through selenoprotein‐mediated pathways. Moreover, selenoproteins modulate the activity of key cellular components such as protein kinases, phosphatases, and transcription factors like NF‐kB by modifying signaling proteins through thiol oxidation [32]. Additionally, selenoproteins play a significant role in the differentiation of adipocytes and enterocytes [33].

Selenium nanoparticles (SeNPs) stand out from other metal nanoparticles such as Ag and Au, which, despite offering several advantages, are more expensive to synthesize. The current study aims to underscore the anticancer effects of SeNPs while shedding light on potential molecular mechanisms underlying their anticancer activity.

## 2 Therapeutic Applications of SeNPs Against Cancer

Cancer stands out as one of the most formidable diseases of the twenty‐first century, evoking significant concern among medical professionals and researchers. The challenge is compounded by the persistent issues of drug‐induced toxicity and resistance, further complicating treatment strategies. In the battle against cancer, a myriad of treatment approaches have been explored and tested. Nanotechnology has significantly bolstered our arsenal against cancer by facilitating tailored treatment strategies, enabling improved targeting while mitigating toxicity. In the relentless fight against cancer, marked by its status as one of the most daunting diseases of our time, medical professionals and researchers grapple with multifaceted challenges [34]. Drug‐induced toxicity and resistance pose persistent hurdles, complicating treatment endeavors [35]. Yet, diverse treatment modalities have been vigorously explored and trialed amidst these challenges. Nanotechnology, a burgeoning field, has emerged as a beacon of hope, empowering tailored treatment approaches that enhance precision targeting and alleviate toxicities. This transformative technology not only strengthens our armamentarium against cancer but also underscores the pressing need for reliable biomarkers to optimize treatment selection and efficacy assessment in the complex landscape of cancer therapeutics [36]. As the quest for biomarkers extends across various cancers, including renal cell carcinoma, advancements in molecular biology offer promise for refining prognostication in the era of immuno‐oncology [37, 38]. Despite ongoing efforts to identify effective biomarkers, validation remains a challenge, emphasizing the imperative for robust markers to guide treatment selection and therapeutic strategies.

*Orthosiphon stamineus* and *Luffa cylindrica* extracts employing green synthesis of Selenium nanoparticles (SeNPs) have great potential for biomedical applications. These SeNPs with techniques such as UV–vis spectroscopy, FTIR, SEM, and XRD revealed that the synthesized SeNPs are stable, crystalline, and exhibit different morphological shapes and sizes of between 30 and 120 nm [39, 40]. They also had low cytotoxicity and less than 5% hemolysis of blood cells means that are suitable for therapeutics, diagnostics, and potentially cardiac tissue engineering. Moreover, the synthesized rutin‐capped silver–selenium nanoparticles (Rut‐AgSeNPs) also exhibited excellent antimicrobial activity against the main dental pathogens and hence revealed the potential nanodrugs in various medical applications [41].

**FIGURE 1:**
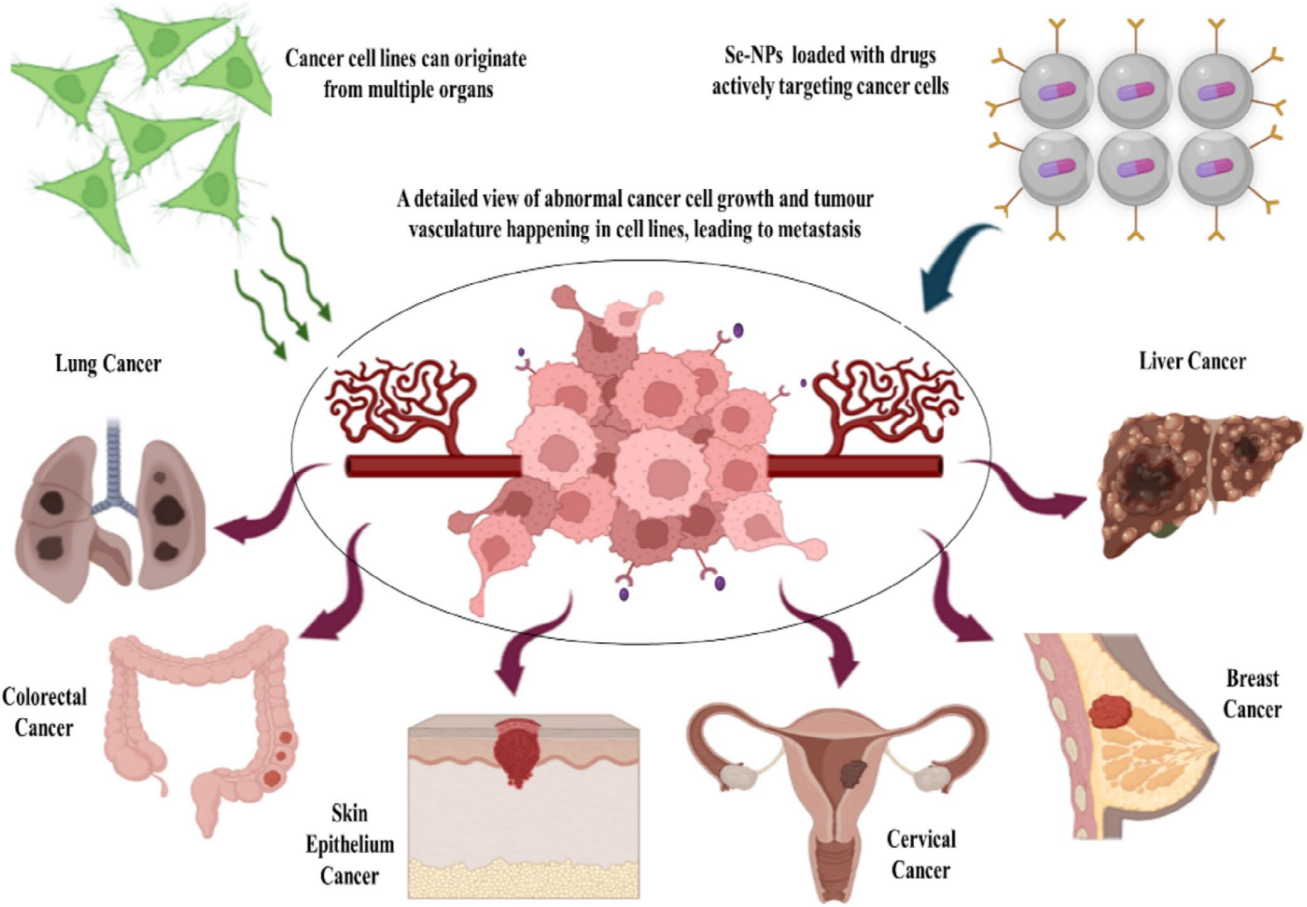
Application of SeNPs on various cancer cell lines.

Among the inorganic nanoparticles utilized in cancer research, selenium nanoparticles (SeNPs) have emerged as a promising candidate, demonstrating efficacy in inducing cytotoxicity in cancer cells. SeNP‐based techniques hold the potential to overcome pharmaceutical resistance and mitigate the toxicity associated with conventional chemotherapy agents. SeNPs serve as excellent carriers for delivering chemotherapy agents precisely to their intended locations. Notably, SeNPs exhibit selective efficacy against malignant cells while sparing normal cells (Figure 1) [42]. SeNPs have garnered attention across various disease contexts due to their superior properties compared to elemental selenium. They enhance bioavailability while concurrently reducing toxicity. The dual prooxidant and antioxidant effects of SeNPs offer diverse avenues for exploration across a spectrum of clinical conditions. This section delves into the multifaceted therapeutic potential of SeNPs, particularly highlighting their extensive anticancer activity both in vitro and in vivo. Table 1 succinctly summarizes SeNPs' anticancer efficacy against various cancer types. (Figure 1) displays the application of SeNPs on various cancer cell lines with reference to its biomedical potential [62, 63].

**TABLE 1:** Anticancerous activity of SeNPs on various cancer cell lines.

Mode of synthesis	Morphology/Structure	Size (nm)	Exposure time	Cancer type	Cell line	IC_50_ value	References
Biological method	Spherical	79.9 nm	48 h	Liver cancer	HepG2	46.8 μg/mL	[43]
Biological method	Spherical	100–250 nm	48 h	Liver cancer	HepG2	50 μg/mL	[44]
Biological method	Monodispersed	113 nm	24 h	Liver cancer	HepG2	19.22 μg/mL	[15]
Biological method	Spherical	12–160 nm	24 h	Liver cancer	HepG2	80 μL	[45]
Biological method	Spherical	2–22 nm	48 h	Liver cancer	HepG2	70.79 μg/mL	[27]
Biological method	Spherical	60 nm	24 h	Liver cancer	HepG2	23.4 ± 2.7 μM	[16]
Chemical method	—	—	—	Liver cancer	HepG2	11.57 ± 3.6 μg/mL	[18]
Biological method	—	203 nm	24 h	Breast cancer	MCF‐7	—	[46]
Chemical method	Rod shaped	60 nm	24 h	Breast cancer	4T1	18 μg/mL	[47]
Biological method	Spherical	80.5 nm	24 h	Breast cancer	MCF‐7	8.37 ± 0.97 μg/mL	[48]
Biological method	—	—	48 h	Breast cancer	MDA‐MB‐231	34 μg/mL	[49]
Biological method	Polygonal	79 nm	24 h	Breast cancer	4T1, MCF‐7	—	[50]
Biological method	Spherical		72 h	Breast cancer	4T1	84.19 μg/mL	[28]
Biological method	Spherical	55.9 nm	48 h	Breast cancer	MDA‐MB‐231	34 μg/mL	[13]
Biological method	—	4–16 nm	—	Breast cancer	MCF‐7	—	[42]
Biological method	Spherical	87 nm	—	Triple‐negative breast cancer	—	19 μg/mL	[51]
Biological method	Hexagonal ring structure	88.89 nm	—	Lung cancer	—	—	[14]
Biological method	—	—	24 h	Lung cancer	A549	80 μg/mL	[52]
Biological method	Spherical	45–90 nm	24 h	Lung cancer	A549	25 μg/mL	[2]
Biological method	—	—	24 h	Lung cancer	A549	15 μg/mL	[53]
Biological method	Spherical	75–200 nm	24 h	Cervical cancer	Hela	—	[54]
Biological method	Spherical	150–350 nm	24 h	Cervical cancer	Hela	—	[55]
Chemical method	Spherical	78 nm	48 h	Cervical cancer	Hela	—	[56]
Biological method	Spherical	53.7 nm	48 h	Cervical cancer	Hela	—	[57]
Biological method	Spherical	5–50 nm	24 h	Cervical cancer	Hela	5.5 μg/mL	[58]
Biological method	Spherical	41 nm	—	Cervical cancer	Hela	—	[59]
Biological method	Spherical	—	24 h	Ovarian cancer	A2780	60.95 μg/mL	[5]
Biological method	Spherical	213.6 nm	24 h	Gastric cancer	AGS	—	[60]
Biological method	Hexagonal	88.89 nm	24 h	Prostate cancer	CaP	46.3% ± 4.4% to 77.2% ± 11.4%	[61]
Chemical method	Spherical	417 nm	24 h	Brain cancer	U87	—	[20]

### 2.1 Liver Cancer

Liver cancer, characterized by its aggressive nature and association with cirrhosis and prolonged liver disease, poses a significant global health challenge [57]. Hepatocellular carcinoma (HCC), the primary form of liver cancer, ranks as the third leading cause of cancer‐related mortality worldwide, the fifth most prevalent disease in males, and the seventh most common malignancy in females [63, 64]. Despite advancements in treatment modalities, liver cancer remains notoriously difficult to manage. Various treatment options, including surgery, medical therapies causing local damage, and liver transplantation, offer potential cures for individuals with early‐stage HCC. However, recurrence of HCC postcurative therapy remains a formidable challenge, with recurrence rates exceeding 70% after 5 years [65]. Current chemotherapeutic and ablative approaches have shown limited efficacy in halting the progression of this aggressive disease [66].

The anticancer potential of selenium nanoparticles (SeNPs) was evaluated using the HepG2 cell line, a model system for studying liver cancer. Results indicated that SeNPs exhibited dose‐dependent cytotoxicity against HepG2 cells, inducing apoptosis as evidenced by a significant increase in the proportion of apoptotic cells [61]. SeNPs derived from *Citrus lemon* demonstrated good stability and potent therapeutic effects against HepG2 cells by inducing apoptosis. Exposure to SeNPs induced oxidative stress and mitochondrial dysfunction, ultimately triggering apoptosis in HepG2 cells [15]. Moreover, SeNPs synthesized using *Morinda citrifolia* exhibited concentration‐dependent reduction in cancer cell viability, with low toxicity confirmed through brine shrimp lethality assay [67]. SeNPs produced from an aqueous extract of *Portulaca oleracea* demonstrated selective cytotoxicity against HepG2 cells, suggesting their potential as an effective anticancer agent with low toxicity to normal cells [27]. Similarly, SeNPs modified with laminarin polysaccharide enhanced apoptosis in HepG2 cells via mitochondria‐mediated pathways, as evidenced by upregulation of proapoptotic markers [16, 68]. Additionally, SeNPs modified with ferulic acid showed promising anticancer effects against HepG2 cells, triggering apoptosis via mitochondrial pathways through the generation of intracellular reactive oxygen species and disruption of mitochondrial membrane potential, leading to activation of caspases 3 and 9 [52]. Thus, SeNPs emerge as a novel and promising anticancer agent, particularly effective against human liver hepatoma, offering potential for further exploration in liver cancer therapy. Furthermore, in vivo experiments using zebrafish models confirmed the inhibitory effects of SeNPs on tumor growth, migration, and angiogenesis.

### 2.2 Breast Cancer

Caspases, a class of cysteine proteases, are hypothesized to play a crucial role in the apoptotic pathway, with caspases 3 and 9 identified as significant regulators of apoptosis in mammalian tissues. Their activities serve as reliable indicators of cytotoxic responses [69]. In vitro studies involving the MCF‐7 cell line demonstrated that selenium nanoparticles (SeNPs) induce apoptosis. Cytotoxicity assessments revealed a dose‐dependent inhibition of MCF‐7 cell growth and survival upon SeNP treatment. Flow cytometry and PCR analysis confirmed apoptosis induction in MCF‐7 cells following SeNP treatment. Mechanistically, SeNPs precisely targeted key apoptotic regulators such as Bcl‐2, Bax, and caspase‐3, leading to the release of cytochrome C from mitochondria into the cytoplasm, culminating in cell death and the induction of permanent DNA damage. Additionally, SeNP‐treated MCF‐7 cells exhibited elevated levels of reactive oxygen species (ROS) and experienced heightened oxidative stress. These findings collectively suggest the potential cytotoxicity of SeNPs in breast cancer therapy [58]. Similarly, another study reported that synthetic SeNPs effectively suppressed the proliferation of MCF‐7 cells in a dose‐dependent manner. Cytotoxicity and apoptotic potential were evaluated using the MTT assay, revealing a dose‐dependent effect. SeNP complexes exhibited potent inhibitory effects on cell viability and antioxidant pathways. These findings underscore the efficacy of SeNPs in scavenging free radicals and their toxicity against the MCF‐7 cancer cell line [53]. In a study investigating the efficacy of selenium nanoparticles (SeNPs) and folic acid‐coated SeNPs on the 4 T1 cell line, both in vitro and in vivo experiments were conducted. Folic acid surface‐coated SeNPs exhibited a more potent antiproliferative effect compared to bare SeNPs. Moreover, folic acid‐coated SeNPs enhanced cell death at a lower IC50 concentration. In vivo data further supported these findings, showing a reduction in tumor growth rate upon administration of surface‐coated SeNPs, possibly attributed to the stimulation of intrinsic apoptosis pathways [59]. Another recent study explored the synthesis of SeNPs coupled with Astragalus Polysaccharide (APS), as well as Ag and Au nanoparticles. APS‐SeNPs demonstrated maximum antitumor activity against the MCF‐7 cell line in vitro, suggesting induction of mitochondrial pathway‐mediated apoptosis and inhibition of late‐stage autophagy in MCF‐7 cells [21]. Similarly, SeNPs synthesized from *Chaenomeles speciosa* exhibited antiproliferative effects against MCF‐7 cells by inducing apoptosis and arresting the cell cycle at the S phase [52, 70]. Currently, three main techniques are employed for producing nanoselenium: radiolysis reduction, sonochemical procedure, and chemical reduction [66, 71]. However, these methods pose various risks, including exposure to radiation, ultrasound, and potentially hazardous chemical reagents. In light of these concerns, green synthesis of nanoparticles has gained attention due to its safety, affordability, and the absence of external chemical reagents for capping and stabilization. To evaluate this approach, SeNPs were synthesized using latex from *Carica papaya* . MTT assay demonstrated the biological characteristics of green‐produced SeNPs, including their efficacy against MDA‐MB‐231 cultured cancer cells. Notably, green SeNPs exhibited significantly greater cytotoxicity against MDA‐MB‐231 cells compared to conventional HBL100 cells [72]. Furthermore, the IC50 value of green SeNPs against MDA‐MB‐231 cells was determined to be 34 μg/mL for 48 h, with no observed cytotoxicity against HBL‐100 cells at concentrations up to 50 μg/mL for the same duration. These findings suggest that administering manufactured SeNPs to MDA‐MB‐231 breast cancer lines may offer enhanced therapeutic efficacy [45]. Another investigation compared the anticancer activity of green and chemically synthesized SeNPs. While chemically synthesized SeNPs exhibited enhanced anticancer activity against 4T1 and MCF‐7 cell lines, they were toxic to normal cells such as NIH/3T3 and HEK293. Consequently, biologically synthesized SeNPs were considered a preferable choice as anticancer agents. Moreover, SeNPs synthesized using the fruit extract of *Vaccinium arctostaphylos L*. demonstrated significant inhibition of 4T1 breast cancer cells [28]. Similarly, plant‐mediated SeNPs derived from an aqueous extract of *Diospyros montana* demonstrated dose‐dependent cytotoxicity against the MCF‐7 cell line [73]. Additionally, SeNPs produced with *Solanum Nigrum* exhibited promising anticancer action against triple‐negative breast cancer and strong antioxidant and antibacterial properties [74]. As a result, green‐produced SeNPs represent appealing, eco‐friendly, and nontoxic options for anticancer medicines, showcasing potential benefits across various applications in cancer therapy and beyond. 4T1 breast cancer cells were evaluated with FA‐modified SeNPs and normal SeNPs [47], it was discussed that normal SeNPs had an IC50 of 80 mg/mL while FA‐SeNPs accomplished the same result at a concentration of 18 mg/mL, indicating a higher performance and antiproliferating activity of ferulic acid combination with SeNPs.

### 2.3 Lung Cancer

An imbalance between cell proliferation and apoptosis poses a significant barrier to the elimination of damaged cells, particularly in the context of cancer. Therefore, a crucial aspect of cancer treatment involves activating apoptotic pathways in tumor cells [58]. The tumor suppressor gene p53 and the caspase enzyme play pivotal roles in regular cell surveillance and malignancy prevention [75]. Consequently, caspases, p53, and Bax are considered essential apoptotic markers in cancer therapy.

In a study assessing the combined effect of selenium nanoparticles (SeNPs) and X‐ray exposure on the A549 cell line, potent cytotoxicity of SeNPs against lung cancer cells was observed via MTT assay, with low toxicity observed in normal cells. Interestingly, the caspase expression was enhanced under X‐ray exposure compared to absence, suggesting a synergistic effect between SeNPs and radiation therapy [14].

Plant‐derived nanoparticles containing beneficial bioactive chemicals such as alkaloids, phenols, flavonoids, saponins, and tannins have demonstrated enhanced activity. SeNPs synthesized using *Mucuna pruriens* exhibited enhanced cytotoxicity against the A549 cell line, with an IC50 value of 80 μg/mL [52]. Similarly, SeNPs synthesized from *Withania somnifera* demonstrated antiproliferative effects against A549 cells [2]. Additionally, SeNPs derived from *Cassia Oleoresin* showed a dose‐dependent increase in cytotoxicity against the A549 cell line, with an IC50 value of 15 μg/mL, indicating substantial destruction of viable cells [58]. These findings underscore the potential of plant‐mediated SeNPs as effective agents in cancer therapy and antimicrobial applications.

### 2.4 Cervical Cancer

Selenium nanoparticles (SeNPs) have demonstrated their ability to induce apoptosis in cancer cells through their prooxidant behavior, which triggers the production of reactive oxygen species (ROS) leading to cell death [32]. To assess this, the cervical cancer cell line, Hela, was exposed to SeNPs synthesized by *Pseudomonas stutzeri* . The study observed cytotoxicity, decreased cell migration, and clonogenic proliferation even at low concentrations as low as 5 μg/mL [58]. In another investigation, the efficacy of SeNPs on Hela and HaCat cell lines was evaluated, revealing dose‐dependent cytotoxicity against Hela cells, with viability as low as 3% at 100 μg/mL, while no toxicity was observed against HaCat cells [53].

Furthermore, hyaluronic acid‐functionalized SeNPs loaded with doxorubicin (HA‐SeNPs‐DOX) were studied both in vitro and in vivo against cervical cancer. These nanoparticles exhibited unique cellular uptake in Hela cells, with faster DOX release in acidic environments. In vitro studies revealed induction of apoptosis and downregulation of cell proliferation in Hela cells through HA‐SeNPs‐DOX, particularly when the Bcl‐2 signaling pathway was activated. In vivo experiments demonstrated tumor reduction and increased cancer cell apoptosis with HA‐SeNPs‐DOX, surpassing the efficacy of free Se‐DOX and DOX alone [59]. A nanocomposite coated with 1,6‐α‐D‐glucan (CPA) was developed to enhance the stability and anticancer properties of SeNPs. In vitro studies on Hela cells showed significant antiproliferative effects, potentially mediated by apoptosis and S phase arrest. Furthermore, ROS overproduction, mitochondrial dysfunction, and caspase‐3 activation were identified as contributing factors to the apoptotic pathway induced by CPA‐SeNPs in Hela cells [49]. The efficacy of SeNPs derived from the cyanobacterium Anabaena against cervical cancer was also investigated, revealing a significant antiproliferative effect against Hela cells, with an IC50 of 5.5 μg/mL. This suggested an increase in the accumulation of cancer cells in the sub‐G1 phase, indicative of apoptosis [49]. Moreover, pectin‐decorated SeNPs demonstrated reduced cytotoxicity against normal cells (RWPE‐1) in vitro, along with increased stability [54].

Finally, chitosan nanoparticle‐based nanocomposites encapsulating presynthesized selenium nanoparticles and 5‐fluorouracil were developed as a novel cancer treatment method. These nanocomposites exhibited strong cytotoxic activity against various cancer cell lines, including HCT‐116, HepG‐2, and MCF‐7, surpassing the efficacy of individual components and demonstrating a synergistic effect that enhances therapeutic potential [76].

### 2.5 Other Cancers

Gastric cancer (GC) stands as the second leading cause of cancer‐related mortality, representing a significant global health concern [6]. Despite advancements in GC treatment, outcomes for affected individuals have not seen significant improvement, necessitating the development of innovative strategies. There is been a surge in interest in nanoparticle‐based drug delivery systems due to their potential advantages over conventional chemical techniques in terms of sustainability, energy efficiency, and cost‐effectiveness. Nanoparticles incorporating elements like gold, silver, copper, zinc, and selenium have shown promise in cancer treatment owing to their favorable pharmacokinetics, tumor‐targeting specificity, minimal side effects, and reduced risk of drug resistance [12, 19, 44, 69]. Selenium, a physiologically active element, is crucial in health maintenance and disease prevention [25]. Selenium nanoparticles (SeNPs) have been found to exhibit higher bioavailability and toxicity compared to both inorganic and organic selenium [32]. In one study, SeNPs derived from silymarin (Si) were characterized and found to significantly increase the cytotoxicity of gastric adenocarcinoma (AGS) cells compared to silymarin alone, while exhibiting no toxicity to normal cells. Molecular analyses revealed that Si‐SeNPs enhanced the production of apoptotic proteins and inhibited the PI3K/AKT/mTOR pathways in AGS cells, suggesting their potential to induce apoptosis and inhibit autophagy [47, 71]. Moreover, SeNPs derived from the root extract of *Kaempferia parviflora* were tested on AGS cells, demonstrating an increase in apoptotic signaling indicators and autophagic flux. Treatment with KP‐SeNP also downregulated the PI3K/Akt/mTOR pathway, further supporting their anticancer effects via apoptosis and autophagy [46, 74].

In ovarian cancer, honey‐derived SeNPs exhibited dose‐dependent apoptotic effects on A2780 cells [5]. Additionally, SeNPs showed considerable cytotoxicity against SKOV‐3 and OVCAR‐3 ovarian cancer cell lines, with reduced metastatic potential observed in vitro and minimal toxicity in vivo [77].

SeNPs have been implicated in triggering cancer cell death by controlling key apoptotic proteins, including the caspase family, p53, and ROS [14]. The introduction of SeNPs to prostate cancer cells resulted in apoptosis and cell cycle arrest, with molecular studies suggesting a role for miR‐16 in enhancing SeNP‐induced apoptosis [61].

Glioblastoma (GBM), the most common and severe primary brain cancer in adults, poses significant challenges in treatment due to medication resistance and poor drug absorption across the blood–brain barrier (BBB) [4, 26, 78]. SeNPs have shown promise in glioblastoma treatment by significantly reducing cell viability in a dose‐dependent manner, indicating their potential to cross the BBB and serve as an alternative therapeutic approach for gliomas [20].

Thus, SeNPs hold immense potential in cancer therapy across various types of cancers, offering a multifaceted approach through apoptosis induction, autophagy modulation, and selective cytotoxicity against cancer cells while sparing normal cells.

Selenium Nps have shown a variety of antitumor and cytotoxic effects on different cancer cell lines, including TMP50‐2 natural polysaccharide and SeNP design (Tw‐TMP‐SeNP) research conducted by Shaojie Zhang et al. [79] that was carried out on cancer cell lines (HepG2, A549, and HeLa). The IC_50_ value for HepG2 lines demonstrated enhanced antitumor activity through SeNPs inclusion, which was reported as 46.8 μg/mL while TMP50‐2 showed only moderate or weak antitumor activity. A study conducted by Suseenthar Ramya et al. [44] showed that SeNPs synthesized from M10A62 actinobacterium, Streptomyces *minutiscleroticus* demonstrated high antiproliferative activity (IC_50_ value) when tested against HeLa, HepG2 cancer cell lines resulting in 50 μg concentration for 99.5% HepG2 growth inhibition and 100 μg concentrations for HeLa cell lines.

Dongxiao Cui et al. [15] demonstrated synthesis of SeNPs that were monodispersed and stabilized and displayed antitumor activity with IC_50_ of 19.22 ± 5.3 μg/mL in different concentrations (5, 10, and 20 μg/mL for 24 h) against HepG2 cells as compared to activity in cell lines without synthesized SeNPs. Another study conducted by Dongxiao Cui et al. [18] discussed the antitumor effects of FA, Se NPs, and FA‐Se using HepG‐2 cells via methyl thiazolyl tetrazolium (MTT) assay, which demonstrated an IC_50_ value of 11.57 ± 3.6 μg/mL. In contrast, the IC_50_ value of Se NPs alone was > 100 μg/mL. This suggested that modified selenium nanoparticles with ferulic acid significantly enhanced their antitumor activity compared to selenium nanoparticles utilized individually. This researcher [16] additionally defined laminarin polysaccharides (LP) decorated selenium nanoparticles (LP‐SeNPs), which demonstrated the cytotoxicity and autophagy through cellular assays against HepG2 cells in different concentrations (10, 20, and 40 μM) for 24 h, the IC_50_ value exhibited was 23.4 ± 2.7 μM, suggesting enhanced cytotoxic activity as compared to normal SeNPs.

M. Nagalingam et al. [45] showed green synthesized SeNPs from *Morinda citrifolia* leaves demonstrated a higher anticancerous activity against HepG2 cell lines when succumbed to different SeNPs concentrations (5, 10, 20, 40, and 80 μL). The IC_50_ value at 80 μL had a higher cytotoxic increase observed with cell viability of less than 60% as compared to different concentrations > 60%.

SeNPs synthesized from *Portulaca oleracea* demonstrated enhanced targeting against HepG2 cell lines with a lesser IC_50_ concentration of 70.79 ± 2.2 μg·mL^−1^ as compared to normal cell lines (WI‐38) with an IC_50_ of 165.5 ± 5.4 μg·mL^−1^ [27].

According to Hamed Amiri et al. [5], in their reported results it was visible that SeNPs‐Honey possessed antiproliferative properties when targeting the A2780 cancer cells. A value of IC50 was observed at 60.95 with 34.85 μg/mL in 24–48 h of starting the treatment procedure. On the other hand, honey displayed no cytotoxicity whatsoever when utilized solely in the treatment.

Green synthesized SeNPs significantly affect the MDA‐MB‐231 cells when compared to the HBL100 cell line. Their IC_50_ was determined to be 34 μg/mL in a testing period of 48 h. The HBL‐100 had no cytotoxic properties at values above 50 μg/mL in 48 h [49].

### 2.6 Toxicity of SeNPs

Selenium nanoparticles (SeNPs) have been perceived to have potential applications in cancer through their ability to have preferential lethal effects toward cancerous cells, however, the issue of toxicity comes into play [80]. It is found that the pathology of SeNPs is dependent on size, dose, and type of synthesized SeNPs that may affect its behavior with biological systems [81]. However, at proper concentrations, SeNPs are nontoxic to healthy cells, unlike other chemotherapeutic drugs [32]. Nevertheless, SeNPs have cytotoxic effect, causing oxidative stress at higher concentrations or longer exposure time and resulting in cell death, specifically apoptosis in normal cells [82]. This oxidative stress is mainly caused by the ability to generate reactive oxygen species (ROS), which works to selectively target cancer cells but may potentially cause damage in normal cells as well [83]. Furthermore, the deposition of SeNPs in the hepatoreal region indicates the potential for SeNPs to exhibit toxic effects in the long run due to pathological deposition in the vital organs, liver and kidneys if removed insufficiently from the body [84, 85]. The biocompatibility and degradation of SeNPs are thus the aspects that need to be well controlled so as to minimize the detrimental consequences. Moreover, SeNP possesses toxic properties that can lead to an immune response depending on the surface chemistry and functionalization of nanoparticles. Although functionalization can improve targeting and minimize off‐target effects, it also creates new issues regarding immunogenicity [86, 87].

## 3 Insight on Mechanism of Action of Selenium Nanoparticles

Selenium nanoparticles (SeNPs) have emerged as one of the most extensively studied nanoparticles in the context of cancer therapy. They exhibit remarkable efficacy against malignant cells by inducing cytotoxicity and showcasing antioxidant properties. Notably, SeNPs have demonstrated the ability to mitigate drug resistance mechanisms and the adverse effects of chemotherapy. Their utility extends to effectively delivering chemotherapeutic agents to target sites, all while exerting distinct effects on tumor cells compared to normal cells [61, 88]. Despite their proven efficacy in reducing tumor burden, the precise molecular mechanisms underlying their action remain elusive. However, several studies have made significant strides in elucidating potential mechanisms through which SeNPs exert their anticancer effects. Below is a succinct overview of each mechanism that SeNPs may modulate, shedding light on their anticancer actions.

### 3.1 Anticancer Activity of SeNPs via Autophagy, ROS, and Chemosensitization

Autophagy, a fundamental cellular process, serves to maintain cellular homeostasis by degrading and recycling damaged proteins and organelles [7]. The intricate involvement of selenium nanoparticles (SeNPs) in regulating autophagy, alongside their capacity to induce apoptosis in specific cancer cell types, has been extensively documented. SeNPs significantly influence autophagosome formation, stimulating the autophagic process by upregulating autophagy‐associated proteins, ultimately leading to cell death [16, 30, 33].

Apoptosis, also known as programmed cell death, constitutes a vital component of the intrinsic genetic machinery governing the growth and defense of multicellular organisms. SeNPs elicit apoptosis through various critical signaling pathways, including generating reactive oxygen species (ROS), downregulating antiapoptotic genes, upregulating proapoptotic genes, and activation of caspases [63].

**FIGURE 2:**
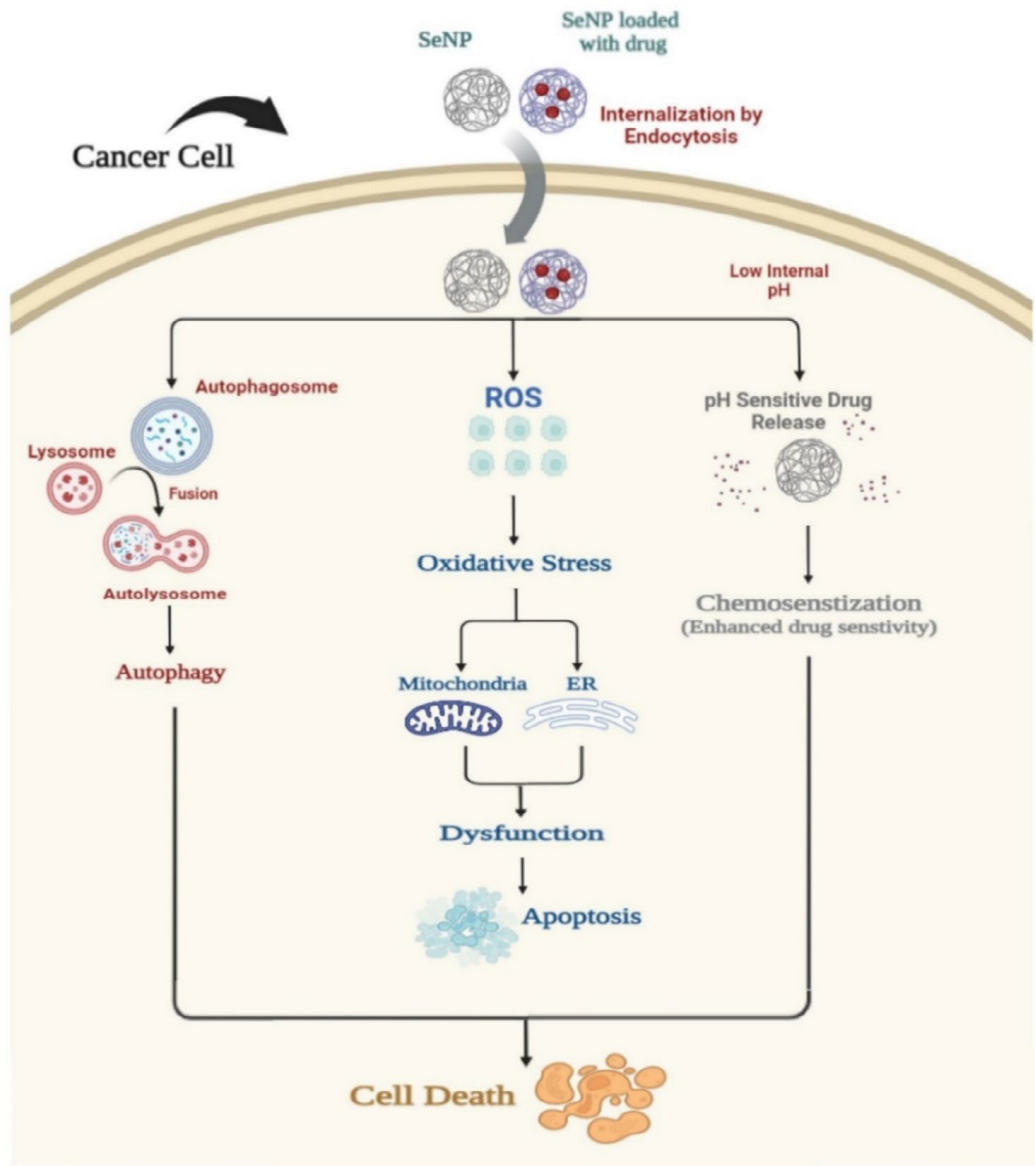
Multiple pathways leading to cell apoptosis through autophagy, production of ROS, and drug‐induced chemosensitization.

The intricate interplay between ROS production and apoptosis, alongside their interactions with the mitochondrial electron transport chain, has been the subject of extensive research. SeNPs induce ROS overproduction, disrupting the mitochondrial electron transport chain and prompting cell death and cell cycle arrest [89, 90, 91, 92]. Drug resistance poses a significant challenge in cancer therapy. SeNPs have demonstrated the ability to enhance drug efficacy, offering a potent route for drug delivery and rendering drugs more responsive to anticancer therapy [29]. Multiple studies have indicated enhanced drug release under low pH conditions, thereby augmenting medication sensitivity in cancer cells (Figure 2) [11, 31, 67].

### 3.2 Anticancer Activity of SeNPs and the Upregulation of mir‐16

MicroRNAs (miRNAs) represent a class of noncoding RNAs pivotal in gene expression regulation by targeting and suppressing the expression of specific genes [93]. While the impact of SeNP therapy on miRNA expression warrants further investigation, recent studies have unveiled a connection between miRNA expression and tumor progression [16]. These microRNA (miRNA) impose significant modulation on the anticancer properties of selenium nanoparticles (SeNPs) across various cancer types. In hepatocellular carcinoma (HCC), SeNPs have been shown to exert influence over miRNA‐7 expression. Notably, the observed reduction in miRNA‐7 levels has been found to correlate with the progression of HCC, indicating a potential mechanism through which SeNPs manifest their anticancer effects in hepatic cancer cells [94]. This relationship underscores the intricate interplay between SeNPs and miRNA networks in the context of HCC pathogenesis. Further elucidation of the molecular details reveals that miRNA‐7 functions as a tumor suppressor by targeting key oncogenes involved in HCC proliferation and metastasis, such as insulin‐like growth factor 1 receptor (IGF1R) and Raf‐1 proto‐oncogene (RAF1). SeNPs, by upregulating miRNA‐7 expression, contribute to the downregulation of these oncogenic targets, thereby inhibiting cancer cell growth and promoting apoptosis.

The antitumorigenic properties of SeNPs in cervical cancer have elucidated involvement in the modulation of miRNA let‐7a expression. The upregulation of let‐7a has been associated with apoptosis induction in cervical cancer cells, shedding light on the epigenetic mechanisms underlying the therapeutic efficacy of SeNPs in cervical cancer management. Molecular investigations reveal that let‐7a exerts its tumor‐suppressive effects by targeting multiple oncogenes, including high mobility group AT‐hook 2 (HMGA2) and cyclin D1 (CCND1) [95]. SeNPs, by enhancing let‐7a expression, effectively suppress the expression of these oncogenic targets, leading to inhibition of cell proliferation and promotion of apoptosis in cervical cancer cells.

In pancreatic adenocarcinoma, SeNPs have shown promise through the inhibition of cell viability, mediated by extracellular vesicles derived from Se‐rich broccoli. This effect has been attributed to the upregulation of miR167a, which mechanistically targets the Insulin receptor within the PI3K‐AKT pathway [96]. Such findings highlight the intricate interplay between SeNPs, miRNAs, and key signaling pathways implicated in pancreatic adenocarcinoma progression. Molecular investigations further elucidate that miR167a suppresses the activation of the PI3K‐AKT pathway by targeting insulin receptor substrate 1 (IRS1), leading to inhibition of cancer cell proliferation and induction of apoptosis.

In Prostate cancer, studies have unveiled the miRNA‐mediated mechanisms underlying SeNPs' inhibition of metastasis. MiR‐155‐5p and miR‐16 have emerged as pivotal regulators, orchestrating the modulation of migration, invasion, and apoptosis in prostate cancer cells [61]. These insights underscore the therapeutic potential of SeNPs in counteracting prostate cancer progression through targeted manipulation of miRNA networks. Molecular analyses demonstrate that miR‐155‐5p and miR‐16 exert their antimetastatic effects by targeting genes involved in epithelial‐mesenchymal transition (EMT) and metastasis, such as matrix metalloproteinase 9 (MMP9) and vascular endothelial growth factor A (VEGFA). SeNPs, by upregulating the expression of these miRNAs, effectively inhibit cancer cell invasion and metastasis in prostate cancer. Recent investigations observed that SeNPs induce the overexpression of miR‐16, resulting in the downregulation of its targets, cyclin D1 and bcl‐2, pivotal in cell proliferation. This mechanism elucidates the apoptotic effects exerted by SeNPs on cancer cells [97].

The collective evidence underscores the pivotal role of miRNA modulation in mediating the anticancer effects of SeNPs across diverse cancer types. Further elucidation of the molecular mechanisms underlying these effects holds immense potential for advancing cancer diagnostics and therapeutics, paving the way for more efficacious and personalized treatment strategies.

One of the primary strengths of the current study lies in its comprehensive exploration of the multifaceted mechanisms underlying SeNPs‐mediated cancer cell death, encompassing autophagy stimulation, oxidative stress induction, and chemotherapy sensitization. By elucidating these intricate pathways, the study provides valuable insights into the potential of SeNPs as a promising cancer therapeutic agent. However, it is essential to acknowledge certain limitations inherent in the study. Firstly, while the in vitro and preclinical evidence presented is compelling, extrapolating these results to clinical settings requires cautious consideration due to potential variations in human responses and pharmacokinetics. Secondly, the precise mechanisms governing SeNPs' toxicity and long‐term effects remain incompletely understood, warranting further investigation. Additionally, the study predominantly focuses on SeNPs' anticancer properties, overlooking potential off‐target effects or unintended consequences. Addressing these limitations through future research endeavors will be crucial for advancing our understanding and utilization of SeNPs in cancer therapy.

### 3.3 Challenges and Limitations

Cancer treatment through selenium nanoparticles (SeNPs) offers a bright hope but it is surrounded by issues and limitation, research milestones, and gaps [98]. One of the difficulties is synthesizing and scaling up a uniform and stable nanoparticle since SeNPs tend to aggregate and degrade [99]. Moreover, includes issues of biodistribution and clearance, with effects of toxicity outside the tumors of interest, and tumor heterogeneity reducing the consistency of action across cancer types [100]. Similarly, toxicity and immunogenicity are considerations that may limit the use of high forces, leading to safety concerns that must be looked at carefully. Promising researchers are involved in the lack of objective biomarkers to determine the rational usage of SeNP, the combination of SeNPs with other treatments, perspectives in clinical applications, and the corresponding regulation and ethical concerns [101]. There is a need to address these issues in order to optimize the SeNPs as a therapeutic agent in cancer treatment. As of the current trend, no distinct clinical trials based on SeNPs have been described, which clearly underlines the requirements to address the in vivo research gap between in vitro experiments and subsequent clinical practice. Future studies should concentrate on the development of particular pathology models, the source of the cell lines, and elaborate studies of the therapeutic application of SeNPs for the treatment of various cancers and other ailments. This will give better direction to clinically applying SeNPs as a potential form of treatment.

## 4 Conclusions

In conclusion, selenium nanoparticles (SeNPs) are a prominent anticancer agent garnering significant attention due to their biocompatibility and ability to interact with biomolecules efficiently. SeNPs exert their anticancer effects through mechanisms such as autophagy induction, oxidative stress generation, and enhancement of chemotherapy sensitivity. Additionally, SeNPs induce oxidative stress, detrimental to cancer cells, and augment the efficacy of chemotherapy. However, concerns persist regarding the potential toxicity of SeNPs in cancer treatment. Further investigation is imperative to delineate the efficacy and safety profile of SeNPs in human trials before widespread adoption in cancer therapy. Nevertheless, the promising prospects of SeNPs in cancer treatment hold transformative potential, offering hope for advancing cancer care paradigms.

## Author Contributions

**Sumaira Anjum and Wisam Nabeel Ibrahim:** conceptualization, writing – review and editing, formal analysis, project administration, and supervision. **Mariam Hashim:** writing – original draft preparation, methodology, and investigation. **Maham Imran:** data curation, writing – original draft preparation, methodology, and visualization. **Sundus Babur:** data curation, writing – original draft preparation, methodology, visualization, and investigation. **Sanniah Adnan:** data curation, writing – original draft preparation, methodology, visualization, and investigation. **Christophe Hano:** resources, validation, and reviewing original draft.

## Conflicts of Interest

The authors declare no conflicts of interest.

## Data Availability

The data that support the findings of this study are available from the corresponding author upon reasonable request.
